#  Maternal self-efficacy and feeding practices in children aged 3-6 years

**Published:** 2015-09

**Authors:** Saeid Doaei, Maryam Gholamalizadeh, Mohammad Hassan Entezari

**Affiliations:** 1 Student’s Research Committee, National Nutrition and Food Technology Research Institute, Faculty of Nutrition Sciences and Food Technology, Shahid Beheshti University of Medical Sciences, Tehran, Iran; 2 Food Security Research Center, Department of Clinical Nutrition, School of Nutrition and Food Sciences, Isfahan University of Medical Sciences, Isfahan, Iran.; 3 Department of Clinical Nutrition, School of Nutrition and Food Sciences, Isfahan University of Medical Sciences, Isfahan, Iran.

**Keywords:** *Lifestyle*, *weight efficacy*, *feeding practi*

## Abstract

**Objective:** Nutrition in childhood has an important role in current and adulthood health. Recent studies have shown that the mother’s lifestyle has an important role in the methods used by mother to feed child. This paper aimed to investigate the association between mother’s weight efficacy lifestyle with feeding practices in children aged 3- 6 years.

**Materials and Methods:** In this cross-sectional study which was carried out in 30 primary schools of Rasht (Iran) in 2012, 165 mothers with children aged 3-6 years were participated. Mothers reported their own and their child’s demographics. Aspects of mother’s weight efficacy lifestyle and mother’s control practices were assessed using Weight Efficacy Lifestyle (WEL) questionnaire and Comprehensive Feeding Practices questionnaire (CFPQ) respectively. Height and weight of mothers participated in the study were measured. The role of mother’s weight efficacy in predicting child’s feeding practices was assessed using linear regression.

**Results:** Results showed that mother’s weight efficacy was related to child feeding practices. The mothers with similar weight efficacy lifestyle applied similar methods in child nutrition. Mothers with better weight efficacy used more encourage balance and variety, environmental control, child involvement and less emotion regulation using foods.

**Conclusion:** **‎** **‏** ‏‎ The result of the ‎study showed that maternal ‎lifestyle was associated with ‎child feeding practices.‎

Eating habits of childhood are continued in adulthood. Thus, malnutrition in this stage brings many problems in adulthood (1). The children nutrition may be impressed by parents, friends, media and personal preferences of children at pre-school ages (2). The influence of parents, who play the role of providers, executors and models for child nutrition at the early stages of childhood, has been recognized as the most important effective factor (3, 4). During the pre-school ages, parents are considered the main individuals in charge for food choices of children and they feed their child using different methods (5).

Studies showed that parents’ life style and their self-efficacy in weight control are effective on the feeding practices and eating patterns of children (3, 6). It seems that based on the important role of life style of mothers in child nutrition and as the behavior change is the most important factor in life style change (7), instead of choosing short-term diets, the eating-based behaviors should be modified in the family and society (8) to mitigate the obesity and other chronic diseases in future.

According to psychology, there are many factors affecting the health-related behaviors including a person belief in his capabilities in applying the change (9). Self-efficacy is defined as one's belief in one's ability to succeed in recognized theories of behavior change as cognitive-social theory of Bandura (10). Bandura theory shows how interpersonal factors such as knowledge and beliefs, social environment and behavior act in a complex relation with each other. This theory emphasizes on the importance of self-efficacy as an intervening factor. Besides cognitive-social theory of Bandura, other models of health behaviors such as health belief models (11) and motivational consulting (12) applied self-efficacy as one of the important factors in behavior change in their theory.

The relation between self-efficacy and food behaviors is investigated from various aspects including the relation of self-efficacy and weight change (13), eating pattern and overweight control (14), lack of eating control (15), fat consumption (16) and fruits and vegetables intake (17). The result of most of the studies showed that self-efficacy is an important factor in prediction of nutrition behaviors.

Weight-efficacy is defined as one’s beliefs about his abilities to resist eating when overeating risk is high (18). Various studies showed that the individuals, who believe in their ability to weight loss, are more successful in this regard (19-22).

To our knowledge, there is not any study that investigated the precise relation between maternal weigh-efficacy and children feeding practices; the present study investigated the relation of maternal weight efficacy life style and child feeding methods**.**

## Materials and Method

***Method***

In a cross-section analytical study, 180 children aged 3 to 6 years from 30 kindergartens of Rasht who were selected through cluster random sampling from the public and private kindergartens were investigated during summer to winter 2012. After visiting the welfare organization of Gilan province (Iran) and the managers of the required kindergartens, the planning was made for face to face visit and data collection from the mothers. In the predetermined day, children who had any form of congenital and metabolic diseases that interact with mother and child’s eating behavior were excluded from this study. The final sample consisted of 208 participants. On that day after talking with the mothers of children, the study aims were explained. Children who had any form of congenital and metabolic disorder that potentially interacted with mother and child’s eating behaviors were excluded and 165 mothers and children were included in the study.

The mothers were asked to complete three questionnaires of general information, Weight Efficacy Lifestyle Questionnaire (WEL) and comprehensive Feeding Practices Questionnaire (CFPQ).

General information questionnaire was applied for data collection of personal characteristics of mothers such as age, education, employment, the number of children, smoking and child’s age, gender and weight at birth. To measure the height and weight of the mother and child, Seca scale and meter were used.

***Maternal self-efficacy***

To study the self-efficacy of the mother regarding the weight control, the validated Weight Efficacy Lifestyle Questionnaire (WEL) was applied (23). The 20-item WEL instrument is consisting of 5-item subscales; the subscales were including the evaluation of mother resistance against eating in five circumstances including food availability in different conditions (e.g., I can resist overeating when I am at a party), negative emotions (e.g., I can resist overeating when I am anxious), social pressure(e.g., I can resist overeating when others are pressuring me to eat) , physical discomfort (e.g., I can resist overeating on the weekend) and positive activities(e.g., I can resist overeating when I am watching TV) Feeding practices 

To study the child feeding method by the mother, comprehensive Feeding Practices Questionnaire (CFPQ) was applied (24)(appendix 1). 

This questionnaire consisted of 46 questions related to child’s control of feeding interactions (four items; e.g., ‘‘Do you allow this child to eat snacks whenever she/he wants?’’), using food to regulate the child’s emotional states (four items; e.g., ‘‘Do you give this child something to eat or drink if she/he is upset even if you think she/he is not hungry?’’), encouraging balance and variety (four items; ‘‘I encourage my child to eat a variety of foods’’), providing a healthy feeding environment (two items; e.g., ‘‘Most of the food I keep in the house is healthy’’), food as reward (three items; e.g., I offer my child his/her favorite foods in exchange for good behavior), child’s involvement in food preparation (three items; ‘‘I involve my child in planning family meals’’), modeling eating behaviors (four items; ‘‘I model healthy eating for my child by eating healthy foods myself’’), monitoring (three items; e.g., ‘‘How much do you keep track of the high-fat foods that your child eats?’’), pressure to eat (four items; e.g., ‘‘My child should always eat all of the food on her plate’’), food restriction for health purposes (four items; ‘‘I have to be sure that my child does not eat too much of his/her favorite foods.’), food restriction for weight control (seven items; ‘‘I have to be sure that my child does not eat too many high-fat foods’’) and teaching about food and nutrition (three items; e.g., ‘‘I discuss with my child why it’s important to eat healthy foods’’). This questionnaire consisted of 46 questions related to child’s control of feeding interactions (four items; e.g., ‘‘Do you allow this child to eat snacks whenever she/he wants?’’), using food to regulate the child’s emotional states (four items; e.g., ‘‘Do you give this child something to eat or drink if she/he is upset even if you think she/he is not hungry?’’), encouraging balance and variety (four items; ‘‘I encourage my child to eat a variety of foods’’), providing a healthy feeding environment (two items; e.g., ‘‘Most of the food I keep in the house is healthy’’), food as reward (three items; e.g., I offer my child his/her favorite foods in exchange for good behavior), child’s involvement in food preparation (three items; ‘‘I involve my child in planning family meals’’), modeling eating behaviors (four items; ‘‘I model healthy eating for my child by eating healthy foods myself’’), monitoring (three items; e.g., ‘‘How much do you keep track of the high-fat foods that your child eats?’’), pressure to eat (four items; e.g., ‘‘My child should always eat all of the food on her plate’’), food restriction for health purposes (four items; ‘‘I have to be sure that my child does not eat too much of his/her favorite foods.’), food restriction for weight control (seven items; ‘‘I have to be sure that my child does not eat too many high-fat foods’’) and teaching about food and nutrition (three items; e.g., ‘‘I discuss with my child why it’s important to eat healthy foods’’). The validity and reliability of the questionnaire were investigated in a separate study and they were verified (25). Items were assessed using 5-point likert scales ranging from ‘‘Never’’ (1) to ‘‘Always’’ (5) or ‘‘Disagree’’ (1) to ‘‘Agree’’ (5). 

Finally, the collected data to describe self-efficacy of the mother regarding weight control by feeding methods were analyzed. The role of mother weight-efficacy in determining the type of the applied approach of the mother for child feeding was evaluated by linear regression analysis. In this study, the effect of some independent variables on many dependent variables is measured; Bonferroni correction coefficient was used to determine significance level of the results so that the results have no difference with uni-variate analysis (26). By this formula, the adjusted significance level for data analysis was 0.003.

## Results

The sample consisted of 102 boys and 63 girls who had a mean age of 4.7 years. The majority of mothers was married (96.4%), employed (81.82%) and had a university degree (60%). Most of the mothers were identified to be overweight or obese (mean BMI 33.22 +/- 3.07).

In terms of the maternal weight efficacy, the mean weight efficacy life style was 52.99. Table 1 provides the raw means (±SD) for the WEL measure.

In terms of the child feeding practices, mean score of any subscale of CFPQ was determined. Higher scores for the feeding scales reflected greater use of scales (27). The frequency that mothers used each feeding practices is shown in table 2. The most commonly used feeding practice was environmental control, encourage to balance, modeling and teaching about nutrition and only a minority used emotion regulation.

Linear regression was used to predict children feeding practices using weight efficacy life style as independent variable. Although our primary analysis was on the WEL total score, values for the subscale scores are included (Table3).

Mothers with better weight efficacy used more encourage balance and variety, environmental control, child involvement and less emotion regulation using foods (p<.0003).

**Table 1:** Describing maternal weight efficacy

**Scale**	**The mean WEL scores(** **±** **SD)**
Total	52.99±11.57
Negative emotions	11.42±3.53
Availability	8.79±3.47
Social pressure	10.36±2.89
Physical discomfort	11.47±2.59
Positive activity	10.95±2.91

**Table 2:** Describing parental control practices (n/%)

	**%**	**N**		**%**	**N**
**Child’s control**			**Modeling**		
Low	6.1	10	Low	0.6	1
Med	60.6	100	Med	21.8	36
High	33.3	55	High	77.6	128
**Emotion regulation**			**Monitoring**		
Low	37.6	62	Low	4.2	7
Med	58.2	96	Med	25.5	42
High	4.2	7	High	70.3	116
**Encourage balance and variety**			**Pressure**		
Low	0	0	Low	23	38
Med	8.5	14	Med	52.7	87
High	98.5	151	High	24.20	40
**Environment**			**Restriction for Health**		
Low	0	0	Low	10.3	17
Med	23	38	Med	64.2	106
High	77	127	High	25.5	42
**Food as reward**			**Restriction for weight control**		
Low	27.9	46	Low	34.5	57
Med	50.3	83	Med	48.5	80
High	21.8	36	High	17	28
**Involvement**			**teaching about nutrition**		
Low	0	0	Low	0	0
Med	10.9	18	Med	15.8	26
High	89.1	147	High	84.2	139

**Table 3:** The role of maternal weight efficacy in child feeding practices

	**Child’s Control**	**Emotion regulation**	**Encourage balance and variety**	**Environmental control**	**Involvement**	**Modeling**	**Monitoring**
	β	β	β	Β	β	β	β
Total	-0.050	-0.213*	1.860*	0.437*	0.203*	0.142	-0.069
Negative emotions	0.161	0.026	0.027	0.036	-0.060	0.070	-0.236*
Availability	0.325*	0.042	0.136	0.080	0.009	0.050	-0.042
Social pressure	0.197	-0.128	0.319	0.073	0.551*	-0.096	-0.064
Physical discomfort	-0.373*	-0.256*	0.173	0.714*	0.119	0.26*	-0.032
Positive activity	0.181	0.156	-0.196	0.045	0.046	-0.23	0.167

* : P<0.0003

## Discussion

The current study aimed to study the maternal weight efficacy life style and the applied methods of the mother in feeding practices of children aged 3 to 6 years. Regarding weight efficacy, the mothers faced with Physical discomfort reported the highest resistance and the lowest resistance against food availability. 

In a similar study done by Chang in Malaysia regarding the study of weight efficacy lifestyle of mother by WEL questionnaire, the mothers were least able to control their eating under social pressure and food availability, according to their WEL score (28).

In the above study, the sample study was of both genders (men and women) with age mean 42 years that is the probable reason of existing differences in the results. A study was conducted by Presnell et al. in USA has emphasized on the role of gender in determining the type of efficacy of an individual in weight control (29). In another study done by Rejeski, the least resistance was reported in eating control under food availability and the results were consistent with the results of the current study (30).The results of the study showed that maternal self-efficacy in weight control was related by the applied methods in child feeding and the mothers with similar weight efficacy lifestyle applied similar methods in child nutrition. In the previous studies the role of maternal self-efficacy and the belief in her ability was supported in weight loss (31-33) and reduction of behavioral disorder of the child (34). Moreover Dottun et al. in a study showed that obese or overweight individuals had lower self-efficacy in weight control compared to others (35). A research done by Sanders, the parents’ self-efficacy was associated with childcare methods (36). In the study conducted by Danaher, it was shown that high self-efficacy of the parents was associated with the improvement of child feeding methods (37).

In the present study, the mother who had less self-efficacy against eating food used a few of the following methods: balance encouragement and variety in eating, environment control and child involvement. The results of the study done by Birch showed that the obese parents applied some methods in child nutrition control leading into child obesity and over weight (38). In our previous study the relation of less application of balance encouragement and involvement with frequent intake of high calorie food was shown (39).

In the present study, the mothers with low self-efficacy in weight control used children emotion regulation by food. In a study done by Gholamalizadeh et al. Mothers obesity was associated with emotion regulation with food (40). In a study done by Swanson, it was reported that low self-efficacy of the mother led into the reduction of the quality of feeding methods and child nutrition (6). Conclusion 

## Limitations

One of the limitations of the present study is the self-report of the mothers regarding self-efficacy in weight control and child feeding methods. Also, this study was cross sectional, which has implications for understanding causality and the relationship between variables. 

## Conclusion

The result of the study showed that maternal weight efficacy was associated with feeding methods of the child. The mothers with more control on their weight applied better methods in child nutrition. The instrumental application of the food was low in these mothers. The longitudinal studies may be more reliable in this regard.

## Appendix1:

**Comprehensive Feeding Practices** **Questionnaire**

1. Do you permit your child to eat whatever he wants?
2. Do you prepare another food for the child in the case he doesn’t like the food you have prepared?
3. Do you permit your child to eat snack whenever he wants?
4. Do you permit the child to leave the dining table when he has had enough, even if the other members of the family are busy eating their food?
5. When the child is seeking excuses, is giving something to drink or eat the first thing you do?
6. When the child is tired, do you give him something to drink or eat even if you suppose he is not hungry.
7. When the child is agitated and stressed do you give him something to drink or eat even if you think he is not hungry?
8. Do you encourage your child to have healthy food instead of unhealthy food.
9. I encourage my child to test new foods.
10. I tell my child that healthy foods taste good.
11. I encourage my child to use foods of different variety.
12. Almost all food that I keep at home are healthy.
13. I Keep large amount of snack such as chips.
14. A variety of healthy food is available at every meal-time to my child.
15. As a reward to my child good behavior I make use of different kinds of sweets. ( candy, ice, cream, cake )
16. As a reaction to my child wrong behavior I for bidden using sweets and dessert.
17. For my child good behavior I give him his favorite food.
18. I keep my child in family food planning.
19. I let my child to help me in food preparation.
20. I encourage my child to accompany me for shopping foods from markets.
21. I set the patterns of a healthy diet for my child by eating healthy food.
22. I try to use healthy foods in front of my child even if I don’t like them.
23. I try to show eagerness to eat healthy food.
24. I show my child how much I enjoy eating healthy food.
25. How much control have you got over using sweets by your child?
26. How much control have you got over using snack by your child?
27. How much control have you got over using fatty food by your child?
28. How much control have you got over using sweet drinks ( soda, juice ) by your child?
29. My child should always eat his food completely.
30. When my child says he is not hungry, I try my best to give him something to eat any way around?
31. When my child eats just small amount of food I try to force him eat more?
32. When my child says he is done eating, I try to persuade him to eat a little bit more?
33. When I don't control my child's diet he eats a large amount of his favorite food?
34. When I don't control my child's diet he eats a lot of low–nutrition food?
35. I have to make sure that my child does not eat more than what is needed from his favorite food.
36. I have to assure that my child does not eat more sweets than is needed.
37. I have to assure that my child does not eat much fatty food.
38. I encourage my child to eat less in order to not get fat.
39. I feed my child in small amounts to control his weight.
40. When my child eats more than usual for?
41. I limit eating foods that might make my child fat.
42. There are some specific foods my child should not eat because they make him fat.
43. I do not allow my child to eat anything between main meals because I don't want him get fat.
44. I discuss with my child why healthy foods are important.
45. I talk with my child about foods nutritional value.
46. Without any explanation I tell my child what to eat and what not to.
